# Editorial: Responses of marine microbes to multiple environmental drivers of global change: The interplay of abiotic and biotic factors

**DOI:** 10.3389/fmicb.2022.975841

**Published:** 2022-09-06

**Authors:** Yuanyuan Feng, Shengwei Hou, Michael Y. Roleda, Fei-Xue Fu

**Affiliations:** ^1^School of Oceanography, Shanghai Jiao Tong University, Shanghai, China; ^2^Shanghai Frontiers Science Center of Polar Science (SCOPS), Shanghai, China; ^3^Department of Ocean Science and Engineering, Southern University of Science and Technology, Shenzhen, China; ^4^State Key Laboratory for Marine Environmental Science, Institute of Marine Microbes and Ecospheres, Xiamen University, Xiamen, China; ^5^Division of Biotechnology and Plant Health, Norwegian Institute of Bioeconomy Research (NIBIO), Ås, Norway; ^6^Biomarine Resource Valorisation, Division of Food Production and Society, Norwegian Institute of Bioeconomy Research (NIBIO), Bodø, Norway; ^7^The Marine Science Institute, College of Science, University of the Philippines, Quezon City, Philippines; ^8^Department of Biological Sciences – Marine and Environmental Biology, University of Southern California, Los Angeles, CA, United States

**Keywords:** global change, multiple stressors, phytoplankton, viruses, bacteria, microbial communities

## Introduction

Global warming and other anthropogenic pressures have led to the ongoing changes in the ocean. These changes are referred to as drivers or stressors, including ocean acidification, surface ocean warming, deoxygenation, and shifts in salinity, density, nutrient, and light regimes (Boyd et al., 2019; IPCC, 2021). The complex matrix of environmental drivers varies at different spatial or temporal scales, thus exerting strong influence on marine microbes inhabiting these environments. On the other hand, structural and functional alternations of microbial communities may provide feedback to the environment and ultimately influences the global climate. It is noteworthy that environmental drivers never act independently in nature, but rather have multiplicative effects on marine ecosystems due to complex stressor interactions (Crain et al., 2008; Boyd and Brown, 2015). Thus, in order to make a more realistic projection of the ecological consequences of global ocean change, it is essential to elucidate the combined effects of environmental forcing on marine life (Boyd et al., 2018).

In addition to the abiotic changes, the interplay between organisms also plays an important role in shaping the geographical distribution of marine life (Wisz et al., 2013; Donelson et al., 2019) and their ecosystem services (Montoya and Raffaelli, 2010). Marine microbial communities, particularly phytoplankton, are among the first responders to complex environmental changes. As the “driving engine” of the ocean, marine phytoplankton have direct impacts on the planktonic marine food web, regulating not only life at higher trophic levels (e.g., protists and zooplankton, etc.; Fox et al., 2018), but also affecting the reproduction and propagation of marine heterotrophs (Ducklow and Carlson, 1992) and viruses (Danovaro et al., 2011). Meanwhile, by decomposing and remineralizing organic debris, marine heterotrophs play an important role in alleviating the nutrient limitation of marine phytoplankton in the oligotrophic ocean (Burkhardt et al., 2014). Thus, interactions between phytoplankton and heterotrophs likely shape the global distribution pattern of marine phytoplankton (Follett et al., 2022). Besides, marine heterotrophs are involved in mediating dissolved organic carbon (DOC) flow to the traditional food chain *via* microbial loop (Azam et al., 1983), and are thought to be responsible to the massive accumulation of recalcitrant DOC (rDOC) in the global ocean *via* microbial carbon pump (Jiao et al., 2010). Therefore, microbial interactions and their feedback to marine environments should be extensively studied in order to better understand the biogeochemical cycles of the future ocean.

**Figure 1:**
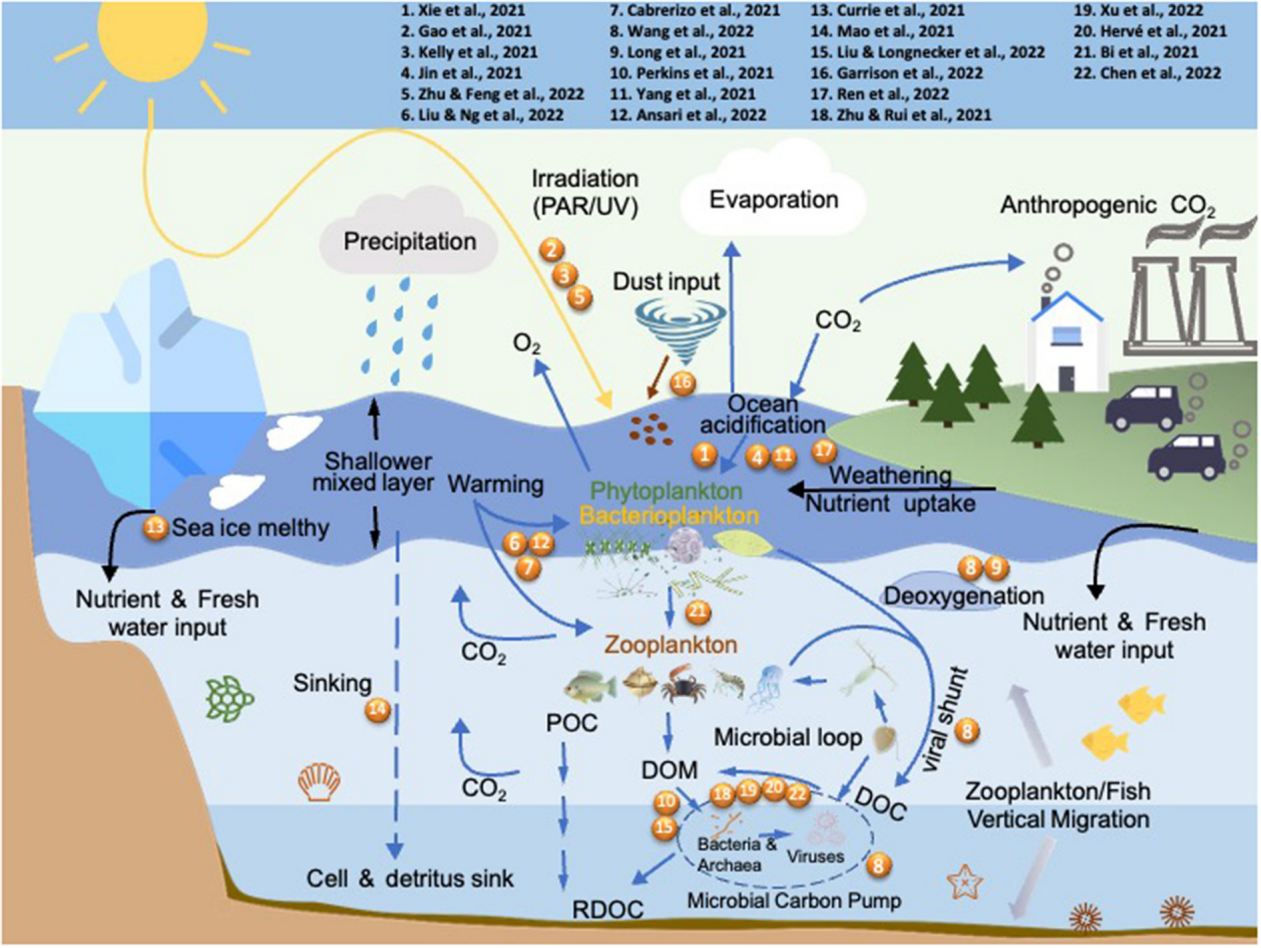
The abiotic and biotic drivers addressed by the published articles in this Research Topic. Numbers refer to the articles listed in the top panel.

This Research Topic summarizes recent advances on the effects of multiple environmental drivers on marine microbes. In total, the authors contributed 22 papers covering a wide variety of subjects including the physiological and molecular characterization of microbial responses to global ocean changes on both single species and community levels, and the depiction of microbial interactions among marine phytoplankton, heterotrophs, and viruses (Figure 1). Across different geographical locations, these studies highlighted microbial responses to environmental changes in the marginal seas of the Northwest Pacific (coasts of China and Viet Nam), the Indian Ocean (Red Sea and the North Indian Ocean), the Atlantic (the Sargasso Sea, coasts of North Carolina and the Caribbean Islands), and the Antarctica. Here we provide an overview of contributions provided in this article collection, as well as perspectives to stimulate future research.

## Contributions

### The effects of multiple environmental drivers on marine microbes

#### Primary producers

Environmental changes may regulate photosynthesis and growth of marine phytoplankton, thus affect oceanic primary production. In this Research Topic, physiological responses of marine phytoplankton to single and multiple environmental drivers (e.g., irradiance, temperature, CO_2_, and nutrient availability) are addressed for marine coccolithophores (Xie et al.) and for marine diatoms (Gao et al.; Kelly et al.; Jin et al.). Coccolithophores are a major calcifying phytoplankton producing calcite scales (coccoliths) in the cell surface (Brownlee et al., 2021). In order to better understand the physiological responses of marine coccolithophores to ocean acidification, Xie et al. dissected the effects of elevated dissolved inorganic carbon (DIC) concentration and reduced pH value on the growth, elemental compositions and cellular macromolecule contents of a model coccolithophore species, *Emiliania huxleyi*. Elevated DIC concentration and reduced pH were observed to synergistically increase the contribution of carbohydrate–carbon to POC, and antagonistically affect the contribution of protein–nitrogen to PON, consequently influencing the C:N ratio and the contribution of *E. huxleyi* to marine biogeochemical cycles. For marine diatoms, previous studies showed that ocean acidification could reduce the dissolution of silica, thus reduced seawater pH may decrease the bioavailability of silica and affect biological silicification (Petrou et al., 2019; Taucher et al., 2022). However, how will other metabolic processes be affected are still not fully resolved. A long-term (~400 days) selection experiment (Jin et al.) examined the lipidomic response of model marine diatom *Phaeodactylum tricornutum* to ocean acidification. Apart from increased production of phosphatidylglycerol (PG) under high CO_2_ conditions (Jin et al.), acidification also triggered substantial lipid remodeling by down- and up-regulating 33 and 42 lipid metabolites, respectively. In addition to ocean acidification, impacts of multiple environmental variables on diatom growth was further examined by Gao et al. and Kelly et al. The synergistic effects of solar ultraviolet radiation (UVR) and reduced CO_2_ availability upregulated the carbon concentration mechanism (CCMs) of a bloom-forming diatom *Skeletonema costatum* (Gao et al.). The results indicate that over the course of bloom when CO_2_ concentration and PAR decrease, UVR helps *S. costatum* promote its carbon fixation, and consequently prolongs the bloom duration. The interactive effects of ultraviolet B (UVB) radiation, warming, and changing nitrogen source on the toxic coastal diatom *Pseudo-nitzschina multiseries* were examined by a multi-variable experimental approach (Kelly et al.). The cellular toxin production of *P. multiseries* were reduced under the warming condition, while increased exposure to UVB could inhibit the bloom formation, indicating that the interactive effects of multiple environmental variables could not be predicted based on simply combining the single variable effects.

Cumulative effects of multiple environmental drivers may differ in various oceanic regions, leading to regional microbial responses. Spatial variations in the photophysiological parameters of the natural *Trichodesmium* populations were observed in both the Northwest Pacific and the North Indian Ocean with a multi-excitation wavelength fast repetition rate fluorometry (FRRf) (Zhu et al.). The regional responses were mainly caused by different nutrient supply and physiological stress in the two study sites. Zhu et al. also suggests that the use of multi-LED generated significant higher values of maximum photochemical efficiency (Fv/Fm) compared to that of blue-only excitation, providing novel insight into photo-acclimation of natural *Trichodesmium* populations.

#### Other microbial groups

The environmental impacts on the primary producers will be passed through the marine food web, and further affect the feeding behaviors of organisms on the higher trophic levels. Liu, Ng et al. observed a selective feeding behavior of the mixotrophic dinoflagellate *Lepidodinium* sp. under different combinations of temperature and nutrient conditions. The higher ingestion rate on high-N prey under lower N supply indicates a compensatory feeding to balance the cellular elemental stoichiometry. On the other hand, warming mainly resulted in an increase in the Chesson selectivity. Apart from mixotrophs, the microzooplankton herbivory is believed to be a major mortality source of marine phytoplankton, playing important ecological roles at the base of pelagic marine food webs (Calbet and Landry, 2004; Caron and Hutchins, 2013). Cabrerizo and Marañon analyzed the thermal sensitivity of grazing pressure by microzooplankton through an extensive literature analysis on paired rate estimates of growth and grazing rates from dilution experiments. Temperature was found to have a highly regional impact on the microzooplankton grazing pressure throughout the world's oceans, with stimulatory effects in polar open-ocean or tropical coastal regions, but inhibitory effects elsewhere. Nitrate availability worked synergistically with temperature in stimulating grazing pressure in open-ocean ecosystems, but antagonistically in coastal environments, particularly in polar regions.

In addition, other groups in the microbial community, including bacteria, archaea, viruses, protists, and fungi are also important components of the marine ecosystems. For example, viruses constitute a great part of marine nitrogen inventory and recent discoveries of novel viruses involved in nitrogen biotransformation processes provide new insights into the marine nitrogen cycling (reviewed by Wang et al.). Long et al. reviewed the current understanding and knowledge gaps of the microbial ecology in the oxygen minimum zones (OMZs). The review covers the formation of OMZ, abiotic and biotic factors affecting OMZ expansion, and the ecology of OMZ microbes and their responses to climate change. Several directions to close the current knowledge gaps were proposed, including the need for a more comprehensive approach, such as *in situ* incubations to characterize microbial responses to climate change, and the call for investigations of the least studied microbial groups in natural microbial communities of OMZs, such as microeukaryotes and viruses. Perkins et al. investigated the potential of endophytic fungi to degrade kelp detritus, a major source of cellulose in marine systems. Their results suggest that endophytic kelp fungi may play a significant role in marine carbon cycling *via* polymeric organic matter degradation under both oxic and anoxic conditions.

#### Microbial communities

Considerable studies have focused on the effects of global change on single marine species, while microbial community responses to environmental changes are still understudied, particularly in the perspectives of community resilience and adaptive evolution (Hutchins and Fu, 2017; Collins et al., 2020). Short- and long-term microbial community-level studies are required for predicting and mitigating the impacts of future climate change on marine ecosystems (Cavicchioli et al., 2019). Microbial communities in several typical ecosystems were assessed in this Research Topic. By conducting a microcosm incubation experiment, Yang et al. reported that the bacterioplankton communities in a coastal ecosystem in the eutrophic Xiamen Bay, South China Sea, could be adaptable to the short-term elevated *p*CO_2_. A high-frequency assessment of bacterial community dynamics in Red Sea coastal waters suggests that temperature, chlorophyll-*a*, and dissolved organic carbon concentration were the major environmental variables affecting bacterial abundance and diversity patterns (Ansari et al.). In the polar regions, climate change especially tends to drive dramatic environmental changes, including the ice coverage. Currie et al. assessed the effects of legacy sea ice conditions on benthic microbial communities in different regions of Antarctica, by comparing the benthic microbial communities underlying first-year and multi-year sea ice. The sea ice-benthic coupling reveals that the sea ice dynamics have crucial influence on the ecosystem functioning in a changing polar environment.

Understanding the responses of microbial communities to physical disturbances is also critical for predicting nutrient cycling and ecosystem stability. In this article collection, Mao et al. showed that microbial community structure may be impacted by mesoscale physical processes. Moreover, deep convective mixing of dissolved and suspended organic matter is an important export pathway in the biological carbon pump. Liu, Longnecker et al. assessed the fate and compositional transformation of dissolved organic matters by marine microbes in the northwestern Sargasso Sea. The biogeochemical and microbial parameters were analyzed on a seasonal timescale from 2016 to 2017, and the results highlight the contribution of microbes in the DOM transformation and export. Furthermore, extreme weather events, such as hurricanes, floods, droughts, wildfires, etc., are expected to increase in frequency, duration and intensity under global warming scenarios (Diffenbaugh et al., 2017). In this regard, Garrison et al. examined the impact of hurricane season on the coastal microbial community dynamics within the barrier island system of Outer Banks, North Carolina. Strong disturbances of planktonic bacterial communities were observed at offshore sites after hurricanes, which favored fast-growing copiotrophs rather than autotrophs, further suggesting enhanced organic matter remineralization and nutrient cycling in future warmer oceans.

Lastly, in order to mitigate the progress of global warming caused by anthropogenic CO_2_ emission, alkalinity enhancement through weathering reactions has been proposed as a potential solution to increase the capacity of CO_2_ absorption by seawater. The impacts of olivine addition on the bacterial diversity and community composition in the surface and bottom seawater of a representative marine ranch area were studied by Ren et al. in the northern coastal area in China. The results suggest that the olivine addition may shift the bacterial community structure, and thus reveal the need for risk evaluation of the potential ocean geoengineering plan.

### The biotic interactions of microbial organisms in a changing ocean

Marine phytoplankton and associated bacteria often establish a close relationship by exchanging metabolites or nutrients, making the microenvironment surrounding phytoplankton cells or colonies a special biosphere, known as the phycosphere (Seymour et al., 2017). How is the phycosphere structured and regulated under environmental disturbance remain largely understudied. *Phaeocystis globosa* (*P*. *globosa*) is a bloom-forming microalgal species that morphologically can be in either solitary cells or large colonies. Colonies are the dominant form during *P*. *globosa* blooms, where algal cells embedded in the balloon-like colony periphery and transparent fluid filled inside (Schoemann et al., 2005). Off the coast of Southeast Asia, *P*. *globosa* could form gigantic colonies (up to 3 cm) (Qi et al., 2004). Two articles in this Research Topic (Zhu et al.; Xu et al.) analyzed the bacterial communities associated with *P*. *globosa* colonies using high-throughput 16S rDNA-based amplicon sequencing. Interestingly, they both revealed that the bacterial communities associated with *P. globasa* were evolutionarily conserved and might play an important role in the ecological success of *P*. *globosa*. Zhu et al. reported that the *P. globosa* intracolonial fluid was inhabited mainly by species of *Balneola* and *Labrezia*, and suggested that this core set of bacterial consortia promote *P. globosa* colony formation during the *P. globosa* bloom off the coast of Viet Nam. On the other hand, Xu et al. compared the shared epibionts of two *P*. *globosa* strains isolated from different locations off the coast of South China and revealed that long-term laboratory cultivation might select for agal growth promoting bacteria, such as diverse N_2_-fixing bacteria.

Floating *Sargassum* rafts are complex ecosystems, providing special habitats for various associated species. Hervé et al. used metabarcoding approaches to examine the differences in both the eukaryotic- and prokaryotic-associated communities from pelagic *Sargassum* rafts present at two different sites on Caribbean Islands. Significant differences in microbial community structure and composition between landing *Sargassum*, the surrounding seawater, and *Sargassum* from inland storage sites were detected. Therefore, these molecular inventories of the micro- and meiofauna communities provide baseline information for further characterization of trophic interactions, algal organic matter decomposition, and nutrient transfers at coastal and inland storage sites.

Environmental changes may shift the species competition in marine ecosystems. Bi et al. took a step forward to address the responses of marine diatom-dinoflagellate competition to the interplay of temperature, nutrient concentration, and N:P ratio. Although diatoms dominated under high nutrient concentrations, there was a competitive superiority of dinoflagellates at the highest temperature and very high nutrient concentrations. N:P ratios had negative correlation with diatoms/dinoflagellates ratios and the lipid biomarkers could provide useful tools as indicators of diatoms/dinoflagellates relative composition (Bi et al.). Additionally, the interactions between different lineages of MGII archaea and phytoplankton in the northern coast of South China Sea were studied by Chen et al. The results based on non-metric multidimensional scaling and cluster analysis provide insights into the multiple interactions between co-existing planktonic archaea and phytoplankton species.

## Perspectives

Overall, the integrated results of this article collection extend our understanding of important aspects of the microbial responses to multiple environmental drivers of global change: from abiotic drivers to biotic interactions, from single driver to multiple driver effects, from short term to long term adaptative responses, and from single species level to microbial communities across a broad temporal and spatial scales. For the future research directions, a number of overarching gaps emerge and we make the following suggestions to fill in the knowledge gaps.

First, improvement of experimental designs should be undertaken to investigate the holistic responses of marine microbes to the global change. This requires approaches to connect the effects of single environmental drivers and the interactions of multiple drivers. On the other hand, future studies should consider the interactions between different components of the microbial community, connecting the direct environmental effects with these indirect biotic effects, as well as their linkages with the upper trophic levels on the ecosystem level. Furthermore, the study subject, the specific research questions, and the locale of the experiment need to be considered prior to designing the experiments for the assessment of multiple driver effects. As such, the online tool Multiple Environmental Driver Design Lab for Experiments (MEDDLE, https://meddle-scor149.org, Boyd et al., 2019) provides a good web-based practice guide to direct these research approaches. It provides online materials and tools for designing single and multi- driver experiments, in order to generate more accurate and statistically robust experimental results for ocean research.

Secondly, advanced culture-independent approaches should be applied to natural microbial communities for a better understanding of *in situ* microbial processes and adaptation mechanisms. The rapid expansion of the meta'omics toolbox has enabled direct investigation of microbial abundance, community composition and dynamics, metabolic activities and microbial products in natural microbial communities. Meta'omics approaches bypass the cultivation step, which are not only crucial for studying microbes that are resistant to cultivation, but also necessary for understanding adaptation mechanisms of extremophiles inhabiting inaccessible environments or microbes with extremely slow growth rates. However, accurate partition of biotic interactions from environmental stressors are also challenging with *in situ* microbial communities, and hypothesis testing on synthetic microbial communities could help to dissect biotic interactions from community responses to global change (Grosskopf and Soyer, 2014).

Last but not least, a concerted effort is required to integrate results generated from laboratory manipulation, *in situ* incubation, and environmental data collection based on long term monitoring, as well as model projections. Laboratory manipulation experiments often provide useful information on the response patterns of organisms to single drivers or driver interactions, as well as molecular mechanisms behind the acclimation or adaptation responses. While *in situ* manipulations help to assess these responses at the ecological scale by including a more integrated microbial community. Furthermore, the model projections based on results of manipulation experiments can be further optimized using data obtained from long-term monitoring efforts. These research approaches together will provide a comprehensive view of how marine biota will respond to the changing marine environment at regional, global, and various temporal scales for policymakers.

## Author contributions

YF and SH designed this Research Topic and drafted the editorial. All authors made substantial, revisions, and intellectual contribution to the work and approved it for publication.

## Funding

This Research Topic was financially supported by National Natural Science Foundation of China (41676160), Tianjin Natural Science Foundation (1JCYBJC22900), and Shanghai Frontiers Science Center of Polar Science (SCOPS) to YF. SH was supported by the MEL Visiting Fellowship of Xiamen University (MELRS2210) and by Guangdong Basic and Applied Basic Research Foundation (2021B1515120080). Support was provided by US National Science Foundation grants OCE 2120619, OCE1851222, OCE 2149837, and California State Proposition 84 funding administered by the University of Southern California Sea Grant to F-XF.

## Conflict of interest

The authors declare that the research was conducted in the absence of any commercial or financial relationships that could be construed as a potential conflict of interest.

## Publisher's note

All claims expressed in this article are solely those of the authors and do not necessarily represent those of their affiliated organizations, or those of the publisher, the editors and the reviewers. Any product that may be evaluated in this article, or claim that may be made by its manufacturer, is not guaranteed or endorsed by the publisher.
